# Mouse Crumbs3 sustains epithelial tissue morphogenesis *in vivo*

**DOI:** 10.1038/srep17699

**Published:** 2015-12-03

**Authors:** Lucie E. Charrier, Elise Loie, Patrick Laprise

**Affiliations:** 1 Département de Biologie Moléculaire, Biochimie Médicale et Pathologie/Centre de Recherche sur le Cancer, Université Laval, Québec, Canada; 2 CRCHU de Québec-axe oncologie, Québec, Canada

## Abstract

The human apical protein CRB3 (Crb3 in mouse) organizes epithelial cell polarity. Loss of CRB3 expression increases the tumorogenic potential of cultured epithelial cells and favors metastasis formation in nude mice. These data emphasize the need of *in vivo* models to study CRB3 functions. Here, we report the phenotypic analysis of a novel *Crb3* knockout mouse model. Crb3-deficient newborn mice show improper clearance of airways, suffer from respiratory distress and display perinatal lethality. Crb3 is also essential to maintain apical membrane identity in kidney epithelial cells. Numerous kidney cysts accompany these polarity defects. Impaired differentiation of the apical membrane is also observed in a subset of cells of the intestinal epithelium. This results in improper remodeling of adhesive contacts in the developing intestinal epithelium, thereby leading to villus fusion. We also noted a strong increase in cytoplasmic β-catenin levels in intestinal epithelial cells. β-catenin is a mediator of the Wnt signaling pathway, which is overactivated in the majority of colon cancers. In addition to clarifying the physiologic roles of Crb3, our study highlights that further functional analysis of this protein is likely to provide insights into the etiology of diverse pathologies, including respiratory distress syndrome, polycystic kidney disease and cancer.

Epithelial tissues partition body compartments and adjust their biochemical composition by sustaining active transport and secretion of selected molecules^1^. This requires the cohesion of epithelial cells through specialized cell-cell adhesion structures such as the *zonula adherens* (ZA), which is a belt-like adherens junction located near the apex of epithelial cells^2^. The transmembrane protein E-cadherin is a core component of the ZA. E-cadherin promotes homophilic cell-cell interactions and is indirectly linked to cortical F-actin through cytoplasmic adaptor proteins, including β-catenin^2^. The cadherin-catenin complex thus contributes to the trans-epithelial actin network that provides resistance to mechanical forces^2^. Contraction of the ZA-associated actomyosin meshwork also shapes epithelial tissues^3^,^4^, thereby contributing to the functional architecture of organs. For instance, specialized epithelial structures such as sacs and tubules are fundamental for the physiology of lungs and kidneys, respectively^5^. In addition to being involved in cadherin-based cell-cell adhesion, β-catenin is a key mediator of the canonical Wnt pathway. Wnt family members are secreted proteins known to play a crucial role in stem cell maintenance, cell proliferation and cell lineage specification in many tissues^6^. The binding of a Wnt ligand to specific members of the Frizzled family of transmembrane receptors leads to the stabilization of cytoplasmic β-catenin, which can ultimately translocate to the nucleus and contribute to the expression of Wnt target genes^6^.

Tight junctions (TJ) sit apical to the ZA and seal the intercellular space in vertebrate epithelial tissues, thereby precluding passive paracellular diffusion^7^. The selective permeability of TJ is mainly provided by members of the claudin family of proteins^7^. Many peripheral cytoplasmic scaffolding proteins contribute to connect actin filaments to TJ. For instance, ZO-1 supports TJ assembly and functionality by bridging the C-terminal tail of claudins to actin^7^,^8^. In addition to claudins, other transmembrane proteins are enriched at TJ to fine-tune the formation and regulation of these occluding junctions, including occludin, JAMs and Crumbs3 (CRB3)^7^,^9^. The latter is an ortholog of *Drosophila* Crumbs (Crb), which is crucial for the establishment and maintenance of epithelial cell polarity^1^,^10^. The polarized architecture of epithelial cells is organized along an apical-basal axis. The apical domain faces a lumen and constitutes an important site of absorption and secretion, whereas the lateral domain spans across the plan of the epithelium and supports crucial adhesive and signaling cell-cell interactions^1^. TJ are established at the interface between these two membrane territories. Finally, the basal domain contains proteins required for anchorage of epithelial cells to the basal lamina. The polarized distribution of transporters and ion channels is crucial for the transport and secretion functions of epithelia. In addition, epithelial polarity proteins contribute to the proper positioning of intercellular junctions and epithelial tissue morphogenesis^11^.

The mammalian genome encodes three Crb orthologs, namely CRB1, CRB2, and CRB3 (mouse Crb1, Crb2 and Crb3)^12^. CRB1 is predominantly expressed in the brain, cornea, and retina^13^,^14^. Mutations in the human *CRB1* or mouse *Crb1* gene cause retinal pathologies^15^,^16^,^17^. CRB2 is co-expressed with CRB1 in the retina, but CRB2 is also found in other organs such as kidneys^18^. Crb2 maintains retinal integrity and is required for gastrulation of mouse embryos^19^,^20^. CRB3 is widely expressed in epithelial tissues^9^,^21^. The *CRB3* gene encodes two isoforms, namely CRB3A and CRB3B. These single-pass transmembrane proteins have identical extracellular and transmembrane domains, but show divergent cytoplasmic tails due to alternative splicing^9^,^22^. CRB3A is mainly localized to the free apical surface and TJ^9^,^21^. The intracellular domain of human CRB3A and mouse Crb3a is highly conserved. It contains a 4.1, Ezrin, Radixin, Moesin (FERM) domain binding motif (FBM) in the juxtamembrane region, and a PSD-95, Dlg, ZO-1 (PDZ) domain binding motif (PBM) at its C-terminal extremity^9^. The cytoplasmic tail of Crb3a binds to the FERM domain of Ezrin^23^, but the functional impact of this interaction remains to be elucidated. The PBM of CRB3A, composed of the amino acids ERLI, binds to the PDZ domain of PALS1 (Protein Associated with Lin-7), which recruits the protein PALS1-Associated Tight Junction (PATJ) into the CRB3 complex^9^,^24^.

CRB3A contributes to the establishment of functional TJ in cultured epithelial cells^21^,^25^,^26^, and is important for establishment of apical-basal polarity. Specifically, CRB3A acts as an apical determinant^26^,^27^, as reported for its fly ortholog^10^,^28^. Similar to CRB3A, PALS1 and PATJ have been implicated in epithelial polarity as well as TJ formation^26^,^29^,^30^,^31^. This suggests that these proteins are crucial mediators of CRB3A functions. This premise is coherent with the fact that the PBM of CRB3A is necessary for apical-basal polarity establishment and TJ biogenesis^25^,^26^. However, cross-interactions of the PBM of CRB3A or PALS1 with the PDZ protein Par6 also contribute to epithelial polarity and TJ regulation^21^,^32^,^33^. Par6 associates with Par3 and aPKC to form a complex traditionally referred to as the Par complex^11^. Par3 and Par6 contribute to the localization and modulation of the kinase activity of aPKC, which preserves apical membrane identity by phosphorylating several critical targets within the polarity protein network^1^,^11^,^34^. CRB3B is associated with spindle poles during cell division, whereas it is localized to the primary cilium in differentiated MDCK cells^22^. Consistent with this specific distribution pattern, knockdown of CRB3B causes cytokinesis and ciliogenesis defects^22^. The cytoplasmic tail of CRB3B ends with an alternate sequence (CLPI), and is thus unable to bind PALS1^22^. However, CRB3B interacts with importin β-1, an association that is important for CRB3B localization to the pericentrosomal region^22^.

Several pieces of evidence suggest that CRB3A may preserve the epithelial phenotype and restrict tumor progression^35^. Loss of CRB3 expression abolishes contact-mediated inhibition of growth and increases the tumorogenic potential of epithelial cells^35^,^36^,^37^. Moreover, CRB3 expression is repressed during epithelial to mesenchymal transition (EMT)^38^,^39^,^40^. EMT is a hallmark of cancer progression that is characterized by loss of epithelial polarity, alteration of cell-cell adhesion and increased cell invasion^41^. Forced expression of CRB3 blocks EMT^40^, and CRB3 limits metastasis formation in mice^36^. Overall, the aforementioned studies emphasize the need for animal models to delineate the function of CRB3 *in vivo*, and to ultimately determine whether CRB3 is a *bona fide* tumor suppressor. Recently, the phenotype associated with the constitutive knockout of mouse *Crb3* was reported^23^. Mutant mice showed epithelial morphogenesis defects, but epithelial cell architecture and TJ integrity were normal to a large extent. *Crb*3 mutants displayed ectopic proteinaceous debris in lungs, and died due to acute respiratory distress soon after delivery. In addition, loss of Crb3 is associated with cystic kidneys and villus fusion in the intestine^23^. Here, we describe a novel conditional *Crb3* mouse knockout model, which confirms that constitutive loss of Crb3 is deleterious for epithelial tissue morphogenesis in lungs, kidneys and intestine. We found that Crb3 is required to preserve apical polarity, but not for TJ integrity *in vivo*. Loss of Crb3 also interferes with the formation of a regular epithelial monoloyer in the intestine, thereby reflecting putative role of Crb3 in ZA dynamics and formation of apical lumens. Finally, we provide novel data showing that Crb3 controls β-catenin levels. Together, our findings indicate that Crb3 supports epithelial tissue morphogenesis and organ physiology *in vivo*.

## Results

### Generation of *Crb3* knockout mice

**Figure 1:**
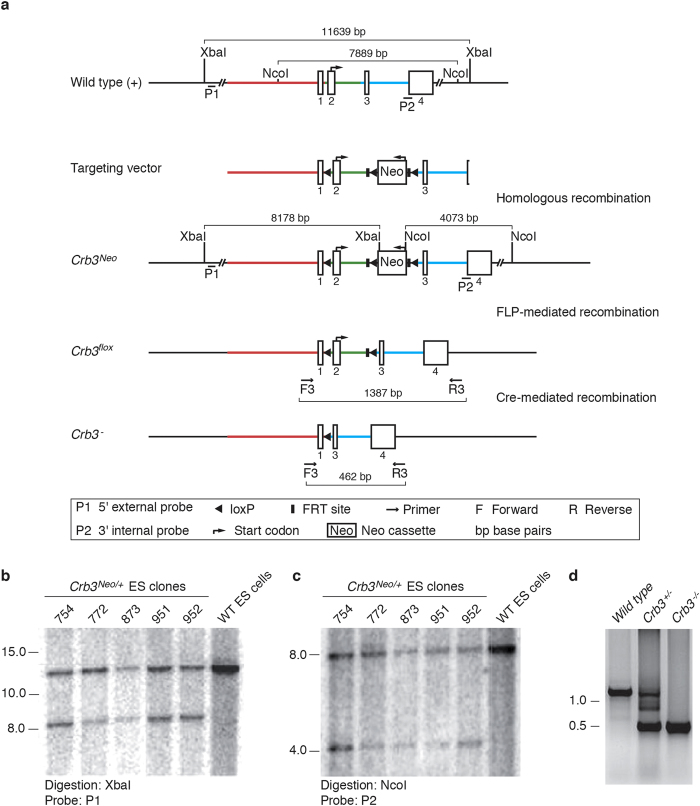
*Crb3* mutant allele. (**a**) Schematic representation of the strategy used to modify the *Crb3* locus and of genotyping approaches. (**b,c**) DNA was extracted from ES clones, digested with XbaI (**b**) or NcoI (**c**) and processed for Southern blotting prior to hybridization with the P1 or P2 probes [see (**a**)], respectively. DNA isolated from wild type (WT) ES cells was used as control. Data confirm homologous recombination at the *Crb3* locus. (d) PCR reaction, amplifying the region contained in-between exon 1 and exon 4 of the *Crb3* gene (F3 and R3 primers; see (**a**) for expected size of amplicons), performed on genomic DNA extracted from E18.5 wild type embryos, *Crb3* heterozygous embryos (*Crb3*^*+/−*^) or *Crb3* homozygous mutants (*Crb3*^−/−^). Cre-mediated recombination efficiently excised exon 2 in mice carrying the *Crb3*^*flox*^ allele.

**Figure 2:**
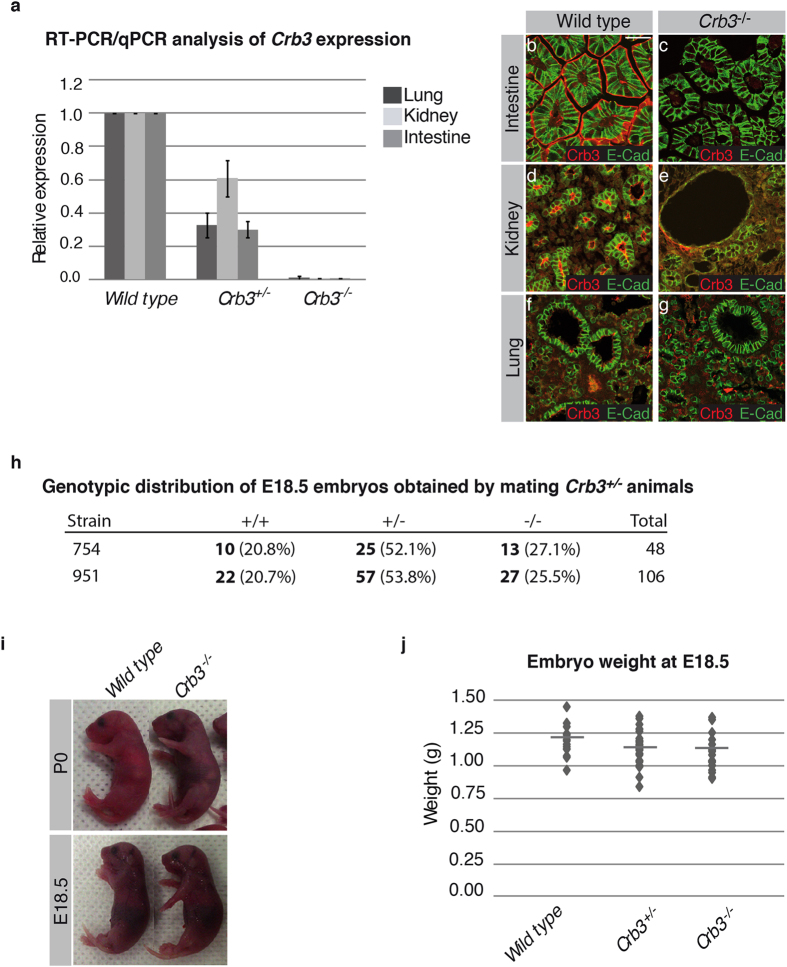
*Crb3* knockout mice do not display gross anatomical defects, but suffer from respiratory distress and die. (**a**) RT-qPCR analysis of *Crb3* expression in the lung, kidney and intestine of E18.5 wild type embryos, *Crb3* heterozygous animals (*Crb3*^+/−^) or *Crb3* homozygous mutant embryos (*Crb3*^−/−^). Primers used amplify exon 2, which contains the sole in-frame start codon. *Crb3* expression was normalized to *actin* levels. (**b**–**g**) Lung, kidney and intestine isolated from wild type or *Crb3*^−/−^ E18.5 embryos were co-stained for Crb3 and E-cadherin (E-cad). Scale bar in b represents 40 μm and also applies to (**c–g**). (**h**) Heterozygous mice of both *Crb3* mutant lines that we established (754 and 951) were mated, and progenies were genotyped at E18.5. The number of mice corresponding to each genotype is written in bold, and the percentage relative to the total number of mice analyzed is in brackets. The percentage of each genotype respects the Mendelian ratios. (**i**) Pictures showing wild type and *Crb3* knockout newborn mice (P0; upper panels) or E18.5 embryos (lower panels). (**j**) Dot plot showing the weight of wild type, *Crb3* heterozygous (*Crb3*^+/−^) or *Crb3* homozygous mutant (*Crb3*^−/−^) embryos at E18.5 (n ≥ 9). The weight of embryos of the different genotypes was not significantly different (ANOVA p ≥ 0.12).

To explore Crb3 functions *in vivo*, we aimed to establish a conditional mouse knockout model. We modified the *Crb3* locus in mouse embryonic stem (ES) cells by homologous recombination using a targeting vector in which exon 2 was flanked by a loxP site and a loxP/FRT Neo cassette (Fig. 1a). Exon 2 is the first coding exon of the *Crb3* gene, and there is no in-frame start codon downstream of it. Thus, Cre-mediated removal of exon 2 is expected to generate a null allele. The XbaI and NcoI restriction sites contained within the Neo cassette were used to discriminate the wild type gene and the recombinant allele (*Crb3*^*Neo*^) by Southern blotting (Fig. 1a–c). Two positive ES clones (754 and 951) were microinjected into blastocysts, and two independent mouse lines capable of germline transmission were obtained. These mice were mated to FLP deleter mice to remove the Neo cassette. Animals carrying the resulting floxed allele (*Crb3*^*flox*^; Fig. 1a) were crossed to a germline Cre deleter mouse line (CMV-*Cre*). Heterozygous mice (*Crb3*^+/−^) devoid of the *Cre* recombinase transgene were then mated to generate knockout animals (*Crb3*^*−/−*^). A PCR reaction amplifying the region comprised between exon 1 and exon 4 confirmed the genomic deletion in heterozygous and homozygous mutant mice (Fig. 1a,d). Consequently, mRNA molecules containing exon 2 were less abundant in *Crb3*^+/−^ heterozygous animals and undetectable in *Crb3*^−/−^ mutant mice (Fig. 2a). Similarly, we observed a loss of Crb3 protein expression in knockout animals (Fig. 2b–g). Together, these data show that we have established a conditional *Crb3* null allele.

### Crb3 is essential for postnatal viability

*Crb3* knockout pups were present at parturition, but show signs of respiratory distress such as arrhythmic breathing, spasms of the chest and cyanosis (Fig. 2i and not shown). We analyzed 158 animals at post-natal day 1 (P1) and found no surviving *Crb3*^−/−^ animals. Thus, loss of Crb3 expression is associated with a fully penetrant perinatal lethality, which likely results from impaired respiratory functions. However, *Crb3*^+/−^ mice were viable and fertile, and showed no obvious phenotype. To circumvent the postnatal viability issue of *Crb3*^−/−^ mutants, we used unborn mice at embryonic day 18.5 (E18.5) to study the impact of Crb3 depletion on general development and epithelial tissue morphogenesis. *Crb3*^−/−^ embryos were represented at the expected Mendelian ratio at E18.5 in both mutant lines that we established (Fig. 2h). Loss of Crb3 expression was not associated with any gross anatomical defects (Fig. 2i), and *Crb3*^−/−^ embryos had a similar weight to their wild type and *Crb3*^+/−^ littermates (Fig. 2j). Overall, these results establish that Crb3 is not necessary for general embryonic development, but is required for postnatal survival.

### Loss of Crb3 is associated with an accumulation of mucosubstances in airways

**Figure 3:**
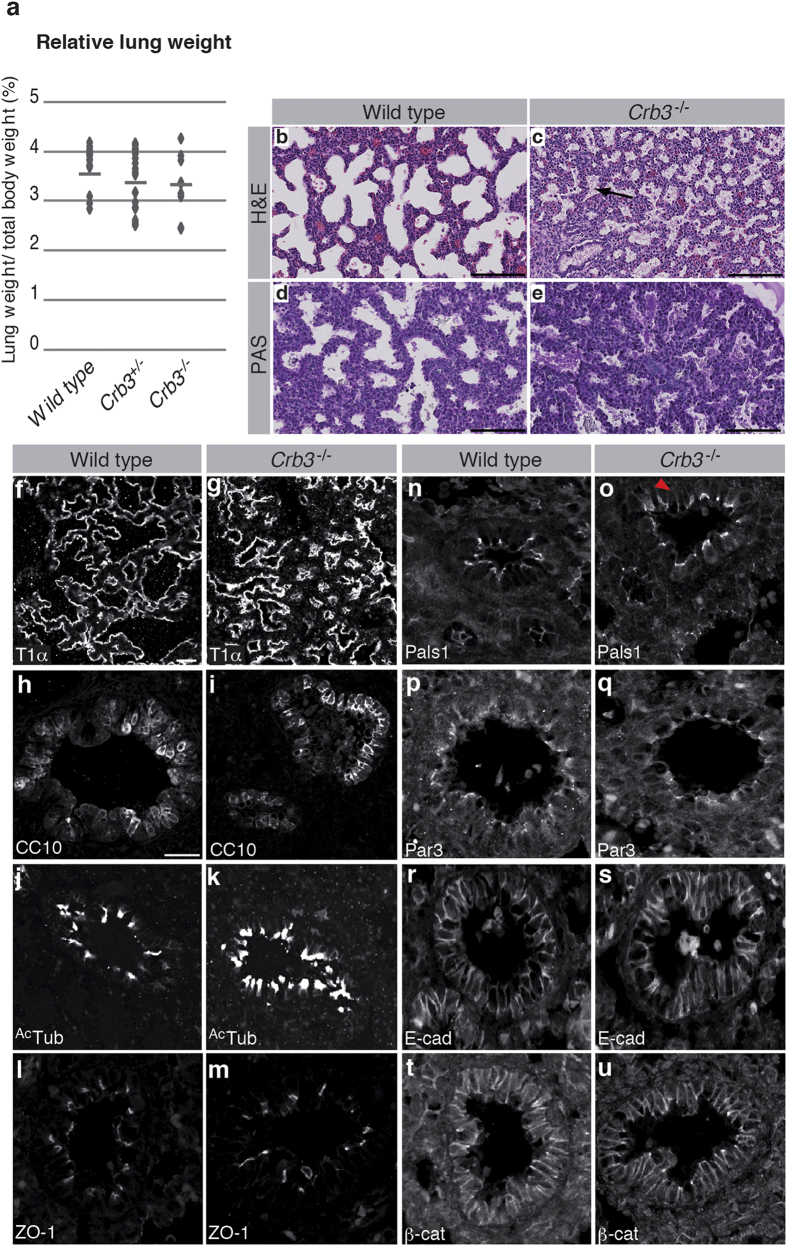
Lungs of *Crb3* knockout mice accumulate ectopic mucosubstances. (**a**) Lungs of wild type, *Crb3* heterozygous (*Crb3*^+/−^) or *Crb3* homozygous mutant (*Crb3*^−/−^) E18.5 embryos were dissected and weighted. Lung weight relative to the total body mass is shown on a dot plot (n ≥ 47), which do not show statistically significant differences among geneotypes (ANOVA p ≥ 0.11). (**b,c**) Paraffin-embedded lungs of newborn wild type or *Crb3*^*−/−*^ mice were sectioned and stained with Hematoxylin and Eosin (H&E). (**d,e**) Paraffin-embedded lungs of E18.5 wild type or *Crb3*^−/−^ embryos were sectioned and stained with Periodic acid–Schiff stain (PAS; (**d,e)**). Scale bars = 100 μm. (**f–u**) Paraffin-embedded sections of wild type or Crb3 knockout (*Crb3*^−/−^) lungs were stained for T1α (**f,g**), CC10 (**h,i**), actetylated-tubulin (^Ac^Tub; (**j,k**)), ZO-1 (**l,m**), Pals1 (n, o), Par3 (**p,q**), E-cadherin (E-cad; (**r,s**)) or β-catenin (β-cat; (**t,u**)). Scale bar in f represents 50 μm and also applies to g, scale bar in h represents 10 μm and also applies to **i–u**.

The respiratory distress associated with loss of Crb3 expression suggests lung morphogenesis defects and/or impaired lung physiology in mutant animals. We dissected lungs from wild type, *Crb3*^+/−^ and *Crb3*^*−/−*^ embryos at E18.5, and observed no significant difference in lung weight (Fig. 3a). Histological analysis revealed that the overall saccular lung architecture was established in the absence of Crb3 (Fig. 3b,c). However, loss of Crb3 expression caused an atrophy of luminal spaces (compare Fig. 3b,c). Moreover, airways were filled with ectopic debris in *Crb3*^−/−^ mutants (Fig. 3c, arrow). These debris were strongly stained by the Periodic Acid Schiff solution (PAS; Fig. 3d,e), which detects polysaccarides, glycoproteins and glycolipids. This suggests that mucus accumulates in airways of *Crb3* knockout animals. Immunostaining of the T1α and CC10 markers revealed the presence of type I pneumocytes and Clara cells, respectively (Fig. 3f–i). Moreover, ciliated cells were present in the lung epithelium of *Crb3*^−/−^ embryos, as shown by acetylated-tubulin staining that highlights cilia (Fig. 3j,k). The presence of these typical lung cell types suggests that Crb3 is not essential for cell lineage specification and differentiation in the epithelial compartment of lungs.

CRB3 is required for proper TJ assembly in cultured cells, as alteration of CRB3 expression causes defects in the distribution of the TJ-associated protein ZO-1^21^,^25^,^26^. However, the sub-cellular localization of ZO-1 was similar in the lung epithelium of control and *Crb3* knockout animals (Fig. 3l,m), thereby suggesting that Crb3 is not necessary for TJ assembly *in vivo*. We next investigated the polarized architecture of epithelial cell in Crb3-deficient animals by looking at the distribution of the Crb3-associated apical protein Pals1. The apical localization of this protein was preserved in lung epithelial cells devoid of Crb3 (Fig. 3n,o). However, we observed a slight but constant ectopic localization of Pals1 at the lateral membrane in the absence of Crb3 (Fig. 3o, arrowhead). This suggests that a saturable compensatory mechanism maintains Pals1 distribution in the absence of Crb3. We also investigated the subcellular localization of Par3, which is a critical member of the Par polarity complex^11^. Members of this complex are concentrated at TJ and show physical and functional interactions with the Crb3 complex^21^,^32^. Par3 displayed a similar distribution in wild type and *Crb3*^−/−^ lung epithelial cells (Fig. 3p,q). Finally, E-cadherin and β-catenin decorated the lateral membrane in *Crb3* mutant lung epithelial cells, as observed in their wild type counterparts (Fig. 3r–u). E-cadherin and β-catenin staining further revealed that Crb3-deficient cells were cohesive and formed a continuous epithelial tissue. This suggests that the integrity of the lateral membrane and cell-cell adhesion are preserved in the absence of Crb3. Overall, these data show that Crb3 is required for clearance of airways, and thus for lung physiology and animal survival. In addition, our data provide evidence that Crb3 is dispensable for formation of adhesion complexes, including TJ. Finally, the apical-basal axis is established in the lung epithelium of *Crb3* mutant animals, although minor polarity defects are observed.

### Crb3 knockout mice develop cystic kidneys

**Figure 4:**
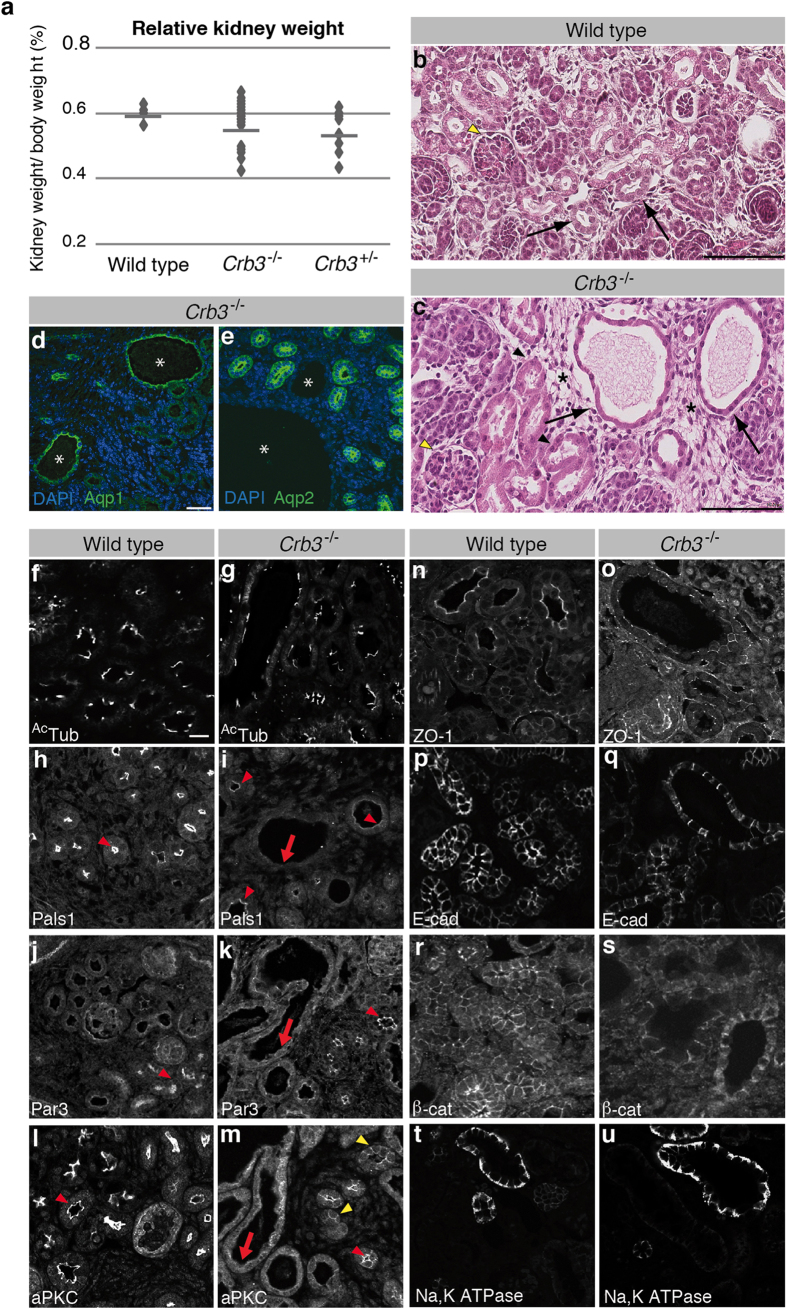
Loss of Crb3 expression is cystogenic in kidneys and impairs apical polarity. (**a**) Kidney weight relative to the total body mass shown on a dot plot (n ≥ 31). Kidney weight is not sensitive to *Crb3* dosage (ANOVA p ≥ 0.33). (**b,c**) Representative images of H&E stained wild type and *Crb3*^−/−^ kidney histological sections. Scale bars = 100 μm. (**d,e**) Immunostaining of Aqp1 (**d**) and Aqp2 (**e**) in *Crb3*^−/−^ embryos (18.5). Asterisks denote cysts. Nuclei were stained with DAPI. Scale bar in d represents 40 μm and also applies to e. (**f-u**) Kidneys were dissected from wild type or Crb3 knockout (*Crb3*^−/−^) E18.5 embryos, paraffin-embedded, sectioned and stained for actetylated-tubulin (^Ac^Tub; (**f,g**)), Pals1 (**h,i**), Par3 (**j,k**), aPKC (l, m), ZO-1 (**n,o**), E-cadherin (E-cad; (**p,q**)), β-catenin (β-cat; r, s) or Na^+^, K^+^ ATPase (**t,u**). Scale bar in f represents 20 μm and also applies to (**g–u**).

CRB3 has been shown to play a role in ciliogenesis in cultured kidney epithelial cells, which display a primary cilium at their apical surface^22^,^42^. Dysfunctions of primary cilia are associated with the pathogenesis of polycystic kidney diseases (PKD), which are characterized by the presence of many fluid-filled kidney cysts and renal failure^43^,^44^. Thus, we investigated kidney morphogenesis in *Crb3* mutant embryos at E18.5. Kidneys from heterozygous and homozygous *Crb3* mutant animals were slightly smaller compared to their wild type counterparts, but the difference in kidney weight was not statistically significant (Fig. 4a). Strikingly, kidneys of *Crb3* knockout embryos displayed numerous cysts lined by cells with a flat morphology, whereas normal tubules are composed of cuboidal cells (compared Fig. 4b,c, arrows). Moreover, a loose interstitial tissue that is typical of fibrosis encountered in renal diseases surrounded cysts^45^ (Fig. 4c, asterisks). Crb3-depleted kidneys also contained some epithelial tubules exhibiting a relatively normal aspect (Fig. 4c, black arrowheads), and glomeruli did not show any obvious defects in the absence of Crb3 (Fig. 4b,c, yellow arrowhead). We stained kidney histological sections of *Crb3*^−/−^ embryos with Aqp1 and Aqp2, which stain proximal tubules and collecting ducts, respectively. Most cysts were positive for Aqp1 and negative for Aqp2 (Fig. 4d,e), showing that proximal tubules are especially sensitive to the loss of Crb3. Overall, this histopathological analysis revealed that loss of Crb3 expression causes a phenotype reminiscent of PKD.

As stated above, PKD is often associated with abnormalities of primary cilium function^42^. However, immunostaining of acetylated-tubulin highlighted a similar number of cilia in wild type and *Crb3* knockout animals (Fig. 4f,g). In addition, normal and Crb3-deprived cells form a primary cilium of comparable length (Supplementary Fig. 1). Cell polarity is also impaired in PKD, and recent evidence suggests that loss of epithelial polarity may contribute to cystogenesis^46^. We thus compared the distribution of polarity markers in wild type and *Crb3*^−/−^ kidney epithelium. The Crb3-associated protein Pals1 is restricted to the apical domain in wild type epithelial cells (Fig. 4h, arrowhead). Pals1 maintained an apical localization in Crb3-deficient epithelial tissues, but showed decreased levels (Fig. 4i, arrowheads). Loss of Pals1 was particularly evident in epithelial cells lining cysts (Fig. 4i, arrow). Consistent with this finding, Crb3-deficient embryos showed a decreased expression level of Pals1 (Supplementary Fig. 2). Similarly, the apical distribution of Par3 and aPKC was preserved in tubules devoid of Crb3 (Fig. 4j–m, red arrowhead), but these proteins were barely detectable at the apical domain of cyst cells (Fig. 4j–m, arrow). Instead, they displayed a cytoplasmic distribution. In addition, aPKC showed ectopic localization at the lateral membrane in the absence of Crb3 (Fig. 4m, yellow arrowheads). The misdistribution of Par3 and aPKC was not associated with a change in their total expression levels (Supplementary Fig. 2), but it indicates important polarity defects in kidney epithelial cells. This contrasts with the subtle misdistribution of polarity markers observed in the lung epithelium (see Fig. 3). We thus investigated the expression and localization of Crb2, which could potentially compensate for the loss of Crb3. *Crb2* mRNA was detected in lungs and kidneys, and its expression levels were comparable in control and *Crb3* mutant embryos (Supplementary Fig. 3a). Crb2 was localized to the apical membrane of lung epithelial cells, whereas the apex of the epithelium lining kidney tubules lacked obvious accumulation of Crb2 (Supplementary Fig. 3b–d). This is consistent with a previous study establishing that *Crb2* mRNA expression is strong in glomeruli, but low or absent in the epithelium of kidney tubules^19^,^47^. We found that Crb2 expression in glomeruli was maintained in *Crb3*^−/−^ embryos (Supplementary Fig. 3h, arrows), and that there is no compensatory upregulation of Crb2 in the kidney epithelium of *Crb3* mutant animals (Supplementary Fig. 3h, arrowheads). Thus, the lack of a putative functional redundancy with Crb2 in kidney tubules could potentially explain why the apical domain of kidney epithelial cells is especially sensitive to loss of Crb3.

In contrast to Pals1, Par3 and aPKC, ZO-1 displayed a similar distribution in kidney epithelial cells of wild type and *Crb3* mutant embryos (Fig. 4n,o). This suggests that the altered distribution of Pals1, Par3 and aPKC results from loss of apical identity or apical differentiation rather than disruption of TJ. We also analyzed the distribution of E-cadherin and β-catenin, which showed a normal lateral distribution in *Crb3* knockout animals (Fig. 4p–s). This suggests that cell-cell adhesion is preserved in the absence of Crb3, and that the identity of the lateral membrane does not require Crb3. The maintenance of baso-lateral membrane identity was further supported by the distribution of the Na^+^, K^+^ATPase pump, which was associated with basal and lateral membranes in both control and Crb3-deficient mice (Fig. 4t,u). Together, these data provide evidence that Crb3 is required to maintain epithelial cell polarity in kidneys. Specifically, Crb3 is essential for the localization of several apical proteins, whereas it has no impact on the distribution of tight and adherens junction components. These polarity defects are accompanied by formation of cysts similar to what is encountered in PKD.

### Loss of Crb3 expression causes villus fusion in the intestine

**Figure 5:**
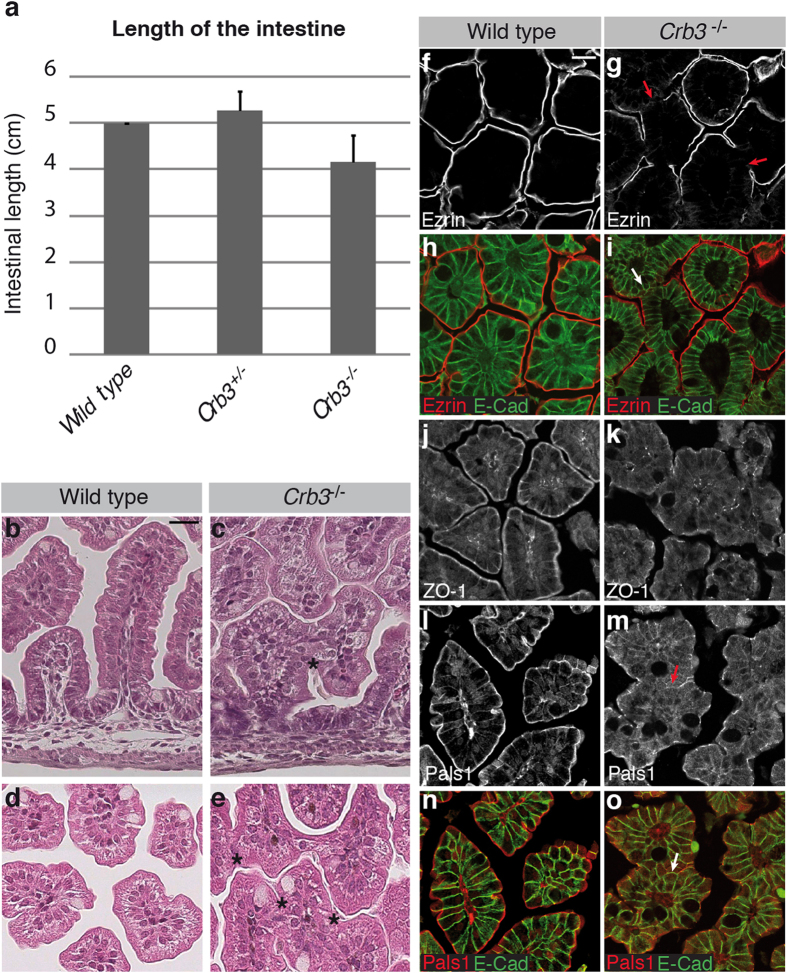
Loss of Crb3 expression results in villus fusion in the intestine. (**a**) Histogram showing the average length of the intestine of wild type, *Crb3*^+/−^ or *Crb3*^−/−^ E18.5 embryos (n = 3). (**b–e**) H&E staining of intestinal histological sections from wild type or *Crb3* knockout (*Crb3*^−/−^) embryos. b and c provide a longitudinal view of villi, whereas d and e depict transversal sections through villi. Asterisks denote fusion points between adjacent villi. Scale bar in b represents 20 μm and also applies to c-e. (**f–o**) Paraffin-embedded intestine of newborn wild type or *Crb3*^−/−^ mice were sectioned and co-stained for Ezrin and E-cadherin (E-Cad; (**f–i)**), stained for ZO-1 (**j,k**), or co-stained for Pals1 and E-cadherin (E-cad; (**l–o**)). Scale bar in f represents 20 μm and also applies to (**g–o**).

To obtain a broader perspective on the role of Crb3 *in vivo*, we analyzed the phenotype of intestinal epithelial cells in *Crb3*^−/−^ embryos. In contrast to the lung and kidney epithelium, the intestinal epithelium is composed of columnar cells. The intestinal epithelium is continuously self-renewing from epithelial stem cells. Proliferative cells are restricted to the inter-villus epithelium in embryos or to the crypt compartment during postnatal life. Differentiated cells are found on villi, which are finger-like structures protruding in the gut lumen. The majority of cells lining villi are absorptive cells, also known as enterocytes. *Crb3*^−/−^ embryos developed an intestine comparable in length to *Crb3*^+/−^ and wild type animals (Fig. 5a). In addition, villi were developed in *Crb3* knockout embryos at E18.5, and enterocytes adopted a columnar morphology similar to wild type cells (Fig. 5b,c). Consistent with the normal architecture of enterocytes, the distribution of the apical marker Ezrin and of the lateral protein E-cadherin was not obviously affected in most cells of the intestinal epithelium of *Crb3* knockout embryos (Fig. 5f–i). Similarly, the TJ protein ZO-1 showed a similar distribution in wild type and Crb3-deficient animals (Fig. 5j,k). However, in the absence of Crb3, Pals1 displayed an ectopic localization in the cytoplasm (Fig. 5l,m). Moreover, while no adhesive contacts were observed between cells of neighboring villi in wild type and *Crb3*^+/−^ mice (Fig. 5b,d and Supplementary Fig. 4), many villi were fused in *Crb3*^−/−^ mouse embryos. Villus fusion created ectopic multilayered epithelial structures (Fig. 5c,e; asterisks), in which epithelial cells were devoid of the apical markers Ezrin and Pals1 (Fig. 5g,m; arrow). In contrast, E-cadherin distribution was apolar in these cells and covered their entire surface (Fig. 5i,o; arrow). Together, these results demonstrate that Crb3 is essential for proper morphogenesis of the intestinal epithelium.

### Crb3 knockout mice display high levels of cytoplasmic β-catenin in the intestine

**Figure 6:**
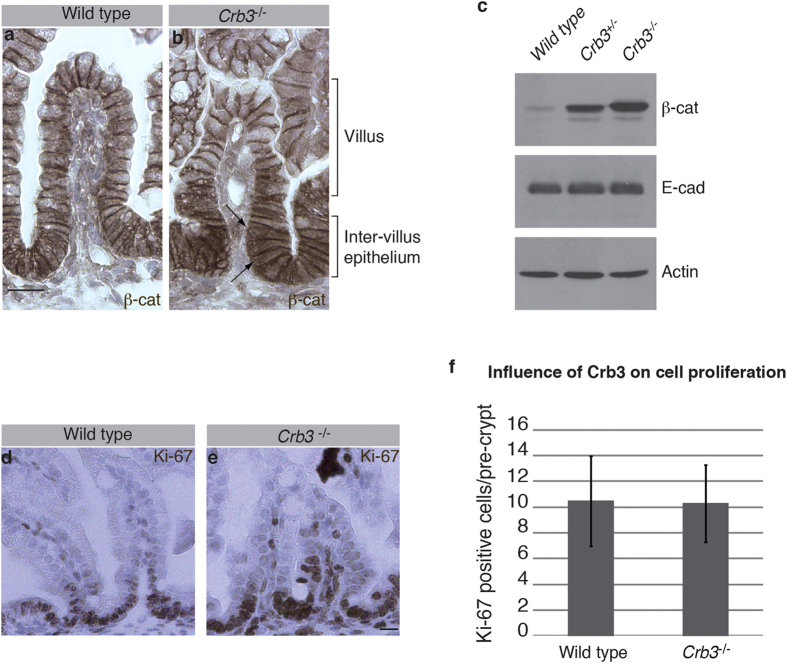
β-catenin expression is up-regulated in the intestine of *Crb3* mutant animals. (**a,b**) Immunohistochemical detection of β-catenin in the intestine of wild type and *Crb3* knockout (*Crb3*^−/−^) E18.5 embryos. Arrows point to nuclei containing β-catenin. Scale bar in a represents 20 μm and also applies to b. (**c**) Western blot analysis of β-catenin, E-cadherin and Actin expression in the intestine of E18.5 wild type embryos, *Crb3* heterozygous embryos (*Crb3*^+/−^) or *Crb3* homozygous mutants (*Crb3*^−/−^). (**d,e**) Detection of the Ki-67 antigen by immunohistochemistry on an intestinal section of a wild type E18.5 embryo or a *Crb3*^−/−^ knockout animal at the same developmental stage. Scale bar in e represents 20 μm and also applies to d. (**f**) Histogram showing the quantification of the number of Ki-67-positive cells. The Y axis represents the number of cells expressing Ki-67 between two consecutive villi. The intestine of wild type and *Crb3*^−/−^ embryos (E18.5) display a comparable number of Ki-67-positive cells (p = 0.93).

Wnt signaling acts as the dominant mitogen for intestinal epithelial cells^6^. Ectopic activation of Wnt signaling drives colon cancer^6^. The importance of this pathway in the digestive track led us to investigate β-catenin distribution in the intestinal epithelium of *Crb3* knockout mice. Consistent with our observation that loss of Crb3 had no impact of E-cadherin distribution outside of villus fusion points (Fig. 5i), β-catenin membrane association was maintained in *Crb3*^−/−^ animals at E18.5 (Fig. 6a,b). In addition, the level of β-catenin in villus epithelial cells was comparable in wild type and *Crb3* knockout animals (Fig. 6a,b). However, the inter-villus epithelium of Crb3-deficient animals showed a strong accumulation of β-catenin as compared to wild type mice (Fig. 6a,b). Although some β-catenin staining was found in the nucleus of *Crb3* mutant cells (Fig. 6b, arrows), β-catenin mostly accumulated in the cytoplasm of these cells. Western blotting confirmed that reduction Crb3 expression caused a dose-dependent increase in β-catenin levels (Fig. 6c).

β-catenin-dependent expression of Wnt-target genes favors cell proliferation, which is confined to the inter-villus compartment at E18.5. As β-catenin expression is mostly increased in this compartment in *Crb3* knockout embryos, we investigated cell proliferation by quantifying the number of Ki-67 positive cells. The number of proliferative cells was similar in the intestinal epithelium of wild type and *Crb3*^−/−^ embryos (Fig. 6d–f). This is consistent with the fact that β-catenin did not show a strong nuclear accumulation in the absence of Crb3 (Fig. 6b). Together, these data demonstrate that loss of Crb3 causes a strong increase in β-catenin expression in the inter-villus epithelium. However, Crb3 deficiency is not sufficient to cause a strong nuclear accumulation of β-catenin or cell proliferation.

## Discussion

In this study, we have established a novel conditional *Crb3* knockout mouse model and showed that ubiquitous loss of Crb3 expression results in respiratory distress and perinatal lethality. Moreover, our data have demonstrated that *Crb3* knockout mice suffer from ectopic accumulation of mucosubstances in lungs, develop cystic kidneys and display fusion of villi in the intestine. Importantly, Whiteman *et al*. also reported these defects using an independent *Crb3* knockout model^23^. While both models allow for a complete loss of Crb3 expression, they result from different modifications of the *Crb3* locus. Moreover, we have used a pure C57BL/6 background, whereas Whiteman *et al*. used a mixed genetic background^23^. Finally, different Cre deleter lines were used to perform germline deletion and to obtain null animals in these studies^23^. Thus, we trust that our phenotypic analysis reveals *bona fide* Crb3 functions. The availability of complementary *in vivo* models will ensure a robust, comprehensive and compelling investigation of Crb3 roles.

It was previously shown that reduction of zebrafish Crb3a expression is associated with a shortening of auditory kinocilia^48^. Similarly, knockdown of CRB3B strongly impairs ciliogenesis in MDCK cells^22^. Surprisingly, our analysis did not reveal obvious changes in the number or length of cilia at the surface of lung and kidney epithelial cells. Lack of strong ciliogenesis defects in lungs may result from a functional redundancy with Crb2, which is expressed in lung epithelial cells (this study and references^18^,^23^). In support of this premise, zebrafish Crb2b is required for renal cilium formation^48^. Although cilia are formed in the absence of Crb3, it is possible that their ultra-structure and/or functions are impaired. Indeed, *Crb3* knockout mice show phenotypes that are suggestive of altered cilia physiology. First, mice devoid of Crb3 display ectopic mucosubstances in airways. This could result from dysfunction of motile cilia, which are required for clearance of the lungs in newborn mice. Of note, zebrafish Crb2b is necessary for cilium motility^48^. Secondly, *Crb3* mutant animals develop cystic kidneys. Several lines of evidence support the notion that cystogenesis results from defective primary cilia^42^,^43^. Thus, as CRB3 localizes to the primary cilium in kidney epithelial cells^22^ and that *Crb3* loss-of-function is cystogenic, the contribution of Crb3 to the integrity and/or physiology of primary cilia would be worth investigating further.

Intracellular signaling pathways initiated by the mechanosensory function of cilia are intimately linked to planar cell polarity (PCP)^44^, which defines the polarized organization of epithelial cells in the plane of the tissue (perpendicular to the apical-basal axis). PCP orients cell division along the longitudinal tubular axis in developing nephrons^44^, thereby ensuring unidirectional tubule elongation. PCP defects randomize the plane of cell division leading to tubule diameter expansion and cyst formation^44^,^49^,^50^. Interestingly, knockdown of CRB3B causes mitotic spindle abnormalities and cytokinesis defects^22^. Moreover, we showed that the expression level of Par3 and aPKC is reduced in Crb3-deficient renal cyst cells, and that aPKC is mislocalized in tubules prior to cyst formation. These defects were never reported before. The Par3-aPKC protein module is required to orient the plane of division in kidney epithelial cells^51^. It is thus possible that alteration of oriented cell division contributes to cystogenesis in *Crb3* mutant embryos. The decreased level of the apical markers aPKC, Par3 and Pals1 also revealed apical-basal polarity defects in renal epithelial cells of *Crb3* knockout animals. This is in agreement with earlier studies in tissue culture showing that CRB3 controls epithelial polarity by acting as an apical determinant^26^,^27^. These observations are also consistent with data from the literature showing a loss of apical-basal polarity in human PKD and animal models of these pathologies^46^,^52^. We have also shown that most cysts develop from proximal tubules in *Crb3*^−/−^ embryos. The morphogenesis of these tubules requires mesenchymal to epithelial transition (MET). This raises the intriguing possibility that Crb3-deficient animals develop cysts owing to improper MET during the formation of proximal tubules. This hypothesis is consistent with previous studies showing that loss of CRB3 expression is associated with an epithelial to mesenchymal transition (EMT) and loss of the epithelial phenotype^36^,^38^,^39^,^40^,^53^. Although additional studies are required to define the mechanisms by which Crb3 maintains the homeostasis and physiology of the kidney epithelium, our data clearly establish that further deciphering of Crb3 functions is crucial for understanding PKD, and perhaps other ciliopathies.

The subcellular distribution of ZO-1 at the apical-lateral border is maintained in *Crb3* mutant epithelial tissues. This suggests that Crb3 is not essential for TJ assembly *in vivo*. This result is in contrast with previous studies showing that alteration of CRB3, PALS1 or PATJ expression leads to TJ defects in cultured epithelial cells^21^,^25^,^29^,^30^,^31^. The role of these proteins in TJ formation was often explored using the calcium switch assay in which cell-cell contacts are first broken by chelating extracellular calcium, and then re-established by addition of fresh culture media. This assay thus reflects early steps of TJ biogenesis. Of note, disruption of the CRB3 complex delays but do not prevent TJ formation after calcium switch^26^,^29^,^30^. Other studies have used overexpression experiments to explore the role of members of the CRB3 complex in TJ integrity^21^,^25^,^26^. These cell culture assays might have revealed a subtle role for CRB3, PALS1 and PATJ in TJ dynamics rather than an essential role for this protein complex in the establishment and maintenance of these junctions. In accordance with this premise, electron microscopy analysis uncovered that Crb3-deficient epithelial cells form TJ in lungs, kidneys and intestine^23^.

Our analysis of the *Crb3* mutant phenotype has highlighted abnormal fusion of intestinal villi. During embryogenesis, the gut is first composed of a stratified epithelium surrounding a central lumen^54^. Cells facing the lumen display a typical apical-basal polarity. In contrast, underlying cells show adhesive contacts all-around their surface and lack a clear apical domain^23^,^55^. Then, this multilayered tissue is transformed to a simple epithelium covering the forming mesenchymal core of villi. This transition results from development and expansion of secondary lumens created by *de novo* formation of apical membrane free of E-cadherin-based cell-cell contacts^55^. The epithelium at fusion points between adjacent villi remains multilayered in Crb3-deficient animals. Moreover, cells composing this ectopic stratified epithelium lack apical markers, whereas E-cadherin is present on the entire surface of these cells. This suggests that Crb3 contributes to apical domain formation and restriction of adherens junction to the lateral domain in intestinal epithelial cells, thereby allowing for the formation of a monolayered and polarized epithelium. Thus, our *in vivo* analysis supports previous studies showing that knockdown of CRB3 in MDCK cells interferes with establishment of polarity and lumen formation^27^,^56^. It was also previously reported that *Drosophila* Crb is require for polarity in renal epithelial tubules only when morphogenetic movements start^57^. Strikingly, in genetic backgrounds where these movements are stalled, polarity is maintained in renal tubules of *crb* mutant fly embryos. This suggests that the primary role of Crb is to maintain the polarity phenotype during cell-cell rearrangements. Thus, the complex cell-cell rearrangements taking place when the intestinal epithelium is converted from a multi-layered tissue to a simple epithelium may explain the critical requirement for Crb3 in this tissue. Interestingly, knockout of mouse *Ezrin* causes a similar intestinal phenotype to the loss of Crb3 expression^55^. Moreover, the cytoplasmic tail of Crb3 interacts with the FERM domain of Ezrin in pulldown assays^23^. This suggests that the physical and/or functional interaction between these two proteins is required for proper morphogenesis of the intestinal epithelium. However, it is also possible that Crb3 and Ezrin act in parallel pathways, as we found that Ezrin is properly localized in the absence of Crb3 in region where villi are not fused. Reciprocally, Crb3 is distributed normally in most cells of the intestinal epithelium of *Ezrin*^−/−^ mice^55^. Concomitant knockout of *Crb3* and *Ezrin* is required to clarify the functional relationship linking the proteins encoded by these genes.

We also reported for the first time that lack of Crb3 expression is associated with a strong increase in cytoplasmic β-catenin levels in the intestinal inter-villus epithelium. This suggests that Crb3 modulates the Wnt signaling pathway, which promotes proliferation in the gut epithelium. In the absence of Wnt, the cytosolic pool of β-catenin is targeted for degradation by the destruction complex, which contains APC. The binding of a Wnt ligand to its transmembrane receptor inactivates the destruction complex and stabilizes β-catenin, which ultimately translocates to the nucleus and contributes to the expression of Wnt target genes^58^. Over 80% of colon cancers result from loss-of-function mutations in the *APC* gene and ectopic activation of the Wnt signaling pathway^6^,^59^,^60^. Although the knockout of *Crb3* is not sufficient to cause nuclear enrichment of β-catenin and excessive proliferation at E18.5, long-term lack of Crb3 expression in the adult intestinal epithelium could favor tumor formation. Indeed, the strong increase in cytoplasmic β-catenin levels in Crb3-deficient cells is likely to act synergistically with genetic alterations supporting nuclear translocation of this crucial mediator of Wnt signalling. Uncoupling of β-catenin stabilization and nuclear accumulation in cancer was supported by analysis of colon cancer samples. Indeed, it was reported that bi-allelic *APC* mutations are associated with accumulation of β-catenin in the cytoplasm, but are not sufficient to cause detectable nuclear translocation of this protein^61^. Moreover, nuclear β-catenin accumulation is observed in an important fraction of intramucosal tumors and invasive cancers but not in adenomas carrying *APC* mutations^62^. These data indicate that defective degradation of β-catenin is required but not sufficient for strong expression of Wnt-target genes, and support a model where molecular alterations other than stabilization of β-catenin contributes to a full activation of Wnt signalling and development of aggressive cancers. Thus, loss of Crb3 expression may prime the development of β-catenin-dependent tumors in the intestine.

Overall, our study revealed that Crb3 is essential for postnatal survival in mice. Our results also support a role for Crb3 in maintenance of the apical domain in epithelial cells. Impaired epithelial cell polarity in *Crb3* knockout mice is associated with epithelial tissue morphogenesis defects reminiscent of human pathologies such as PKD. Moreover, our study provides evidence that Crb3 limits accumulation of cytoplasmic β-catenin in the intestine, thereby suggesting that Crb3 dysfunctions could predispose to colon cancer. Thus, in addition to providing crucial insights into the function of Crb3 *in vivo*, the availability of our mouse model to the research community will contribute to the understanding of pathologies associated with polarity defects, including cancer and renal failure.

## Methods

### Generation of the *Crb3* mutant allele

The targeting vector was constructed from a C57BL/6 BAC clone (RP23: 209N12). The target region contains exon 2 and has a size of 768 bp. The long homology arm extends 6.0 kb 5′ to the single LoxP site, which is inserted upstream of exon 2. The short homology arm extends 2.2 kb 3′ of the LoxP/FRT-flanked Neo cassette, which is inserted in intronic sequences downstream of exon 2. Linearized vector (NotI) was transfected by electroporation in C57BL/6N embryonic stem cells. After selection with G418, surviving clones were analyzed by Southern blots and PCR. Two positive clones (754 and 951) were microinjected into Balb/c blastocysts. Resulting chimeras were mated with B6.Cg-Tg(ACTFLPe)9205Dym/J mice (Jackson Laboratory stock number 005703) to remove the Neo cassette and obtain two lines, originating from two independent ES clones, in which exon 2 is floxed by loxP sites (*Crb3*^*flox*^ mice). Exon 2 contains the start codon, and there is no in-frame ATG codon downstream of exon 2. Thus, removal of exon 2 creates a null allele. *Crb3* mutant mice were generated at inGenious Targeting Laboratory.

### Southern blotting

Genomic DNA was digested with XbaI, submitted to electrophoresis on a 0.8% agarose gel and transferred to a nylon membrane. Digested DNA was hybridized with a probe targeting the 5′ external region of the *Crb3* locus (probe P1, see Fig. 1a). Primers used to generate the P1 probe were as follow: 5′-AGT CTT GGC CCA GAA CTC CTG AG-3′ and 5′-AGT CAG ACC CTA TCT CAG ATA TGC C-3′. An additional strategy was employed to detect the 3′ internal region of the *Crb3* gene on genomic DNA digested with NcoI. The probe used was generated with the following primer pair (probe P2, see Fig. 1a): 5′-TCA GAA GCT CGG ACA CTC ATA GTG-3′ and 5′AGT CCC AGG CGT TGG TAG TGA TG-3′.

### Husbandry and generation of *Crb3* null animals

*Crb3*^*flox*/+^ mice were mated to the B6.C-Tg(CMV-cre)1Cgn/J line (CMV-cre; Jackson Laboratory stock number 006054). CMV-cre is active in the germline. Animals carrying the floxed allele and the Cre recombinase were crossed to C57BL/6J mice. Resulting *Crb3* heterozygous males and females (*Crb3*^+/−^) devoid of Cre recombinase were then bred to generate null animals (*Crb3*^−/−^). Wild type and heterozygous littermates were used as controls. Experiments involving animals were approved by the CRCHU de Québec/Laval University animal care committee, and were carried out in accordance with the approved guidelines.

### Genotyping

Genotyping was performed by PCR (Taq polymerase, Invitrogen) on genomic DNA extracted from tail biopsy specimens by proteinase K digestion (10 mg/ml, 16 h at 55 ^o^C) followed by standard phenol-chloroform extraction, DNA precipitation with isopropanol and DNA resuspension in TE buffer (10 mM Tris, pH 8.0, 1 mM EDTA). Primer pairs used to genotype the *Crb3* locus are illustrated in Fig. 1a and Supplementary Fig. 5). The presence of the distal loxP site was detected with the F1 (5′- GGC ACC TGA GTC TGC ATA GTT GAG AG -3′) and R1 (5′- GTT ATC ACT TAC ACA CCA CTC AGT CAG -3′) primers, whereas the F2 (5′- ATC TGG AGC GAC CGG GTC AAG -3′) and R2 (5′- TTC CTA CCG CAT CTT GGG TCG -3′) primer pair was used to detect the proximal loxP site. Deletion of exon 2 was confirmed using the F3 (5′- ACT AAA CTT TCC GGG TCA CGT G -3′) and R3 (5′- GAA TAG ATG GCT TCC AGA ACC -3′) primers. The *Cre* transgene was amplified using the following primer pair: 5′- CAT TTG GGC CAG CTA AAC AT -3′ (forward) and 5′- CCC GGC AAA ACA GGT AGT TA -3′ (reverse).

### Histology

Dissected organs were fixe in 4% paraformaldehyde (dissolved in PBS) for 2 h at 4 ^o^C, soaked in ethanol 70% and embedded in paraffin. Tissues were then sectioned (5 μm thick) and applied on glass slides. Tissue sections were deparaffinized in xylene (15 min) and rehydrated (successive incubation (3 min) in 100%, 90%, 70% and 50% ethanol solution followed by incubation in distilled water). For hematoxylin and eosin staining (H&E), tissues were incubated in hematoxylin solution (1%) for 5 min, rinsed with water, and successively dipped in 50%, 70% and 90% ethanol solutions. Next, tissues were soaked in eosin solution (1%) for 15 ec, dehydrated with 100% ethanol for 4 min, incubated in xylene for 10 min and then mounted in MM24 Mounting Media (Leica).

### Immunofluorescence (IF) and immunohistochemistry (IHC)

Tissues were fixed, embedded, deparaffinized and hydrated as described above. Antigen retrieval was achieved by boiling specimens for 18 min in a 10 mM citric acid solution (pH 6.0) and allowed to cool down for 30 min at room temperature (RT). Tissues were then washed and permeabilized with a solution of 0.1% Triton X-100 in PBS (PBT) for 10 min. For IHC, endogenous peroxidase activity was blocked with a solution of 3% hydrogen peroxide for 15 min. Then, specimens were saturated with PBT (IF) or PBS (IHC) containing 3% of bovine serum albumin (BSA) for 1 h at RT, or for 20 min with the blocking solution provided with the IDetect Super Stain System (Labs Biotechnology). Primary antibodies were diluted in PBT (IF) or PBS (IHC) containing 0.3% BSA and incubated overnight at 4 ^o^C. Tissue sections were then washed 3 times for 5 min with PBT (IF) or PBS (IHC), and incubated with secondary antibodies diluted in PBT-BSA 0.3% (IF) or PBS-BSA 0.3% for 1 h at RT. For IHC, secondary antibodies were coupled to horseradish peroxidase (HRP), and the antigen-antibody interaction was revealed using the IDetect Super Stain System (Labs Biotechnology) according to manufacturer’s instructions. Nuclei were counterstained with hematoxylin. For IF, secondary antibodies were conjugated to Cy3 (Jackson Immunoresearch Laboratories), Alexafluor 488 (Molecular Probes) or Alexafluor 647 (Jackson Immunoresearch Laboratories). Nuclei were stained with DAPI (1 μg/ml; Roche) for 5 min at RT. Finally, tissues were washed 3 times with PBT (IF) or PBS (IHC) and once with distilled water, and coverslips were mounted using Vectashield (Vector Laboratories). Confocal images were acquired using a confocal microscope (FV1000; Olympus) using a 40× Apochromat lens with a numerical aperture of 0.90. Images were analyzed and processed uniformly using ImageJ (National Institutes of Health) or Photoshop (Adobe). Primary antibodies used were: mouse anti-acetylated alpha tubulin (1/100, Abcam), mouse anti-E cadherin (1/500, Invitrogen), rabbit anti-β-catenin (1/500, Sigma), mouse anti-ZO-1 (1/100, Invitrogen), mouse anti-Ki-67 (1/200, Leica), rabbit anti-PATJ (1/100, Abcam), Syrian hamster anti-T1α pneumocytes (8.1.1; 1/100, DSHB), goat anti-CC10 (gift from G. Singh, USA), rabbit anti-PALS1 (1/100, Santa Cruz Biotechnology), mouse anti-AQP1 (1/200, Santa Cruz Biotechnology), goat anti-AQP2 (1/200, Santa Cruz Biotechnology) and rabbit anti-Crb3 (D2; kindly provided by A. Le Bivic^21^).

### Western blotting

Mouse organs were rinsed with PBS, homogenized with a glass homogenizer in ice-cold lysis buffer (40 mM Tris-HCl, pH 7.6, 1% Triton X-100, 40 mM β-glycero-Phosphate, 100 mM NaCl, 1 mM EDTA, 5% glycerol, 50 mM NaF, 0.1 mM sodium orthovanadate, 0.1 mM phenylmethylsulfonyl fluoride, 10 μg/ml aprotinin, 10 μg/ml leupeptin, and 0.7 μg/ml pepstatin), and processed for SDS-PAGE and Western blotting as previously described^63^. Primary antibodies used were: mouse anti-E-cadherin (1/4000, Invitrogen), rabbit anti-β-catenin (1/4000, Sigma), mouse anti-Actin (1/10000, Millipore), rabbit anti-aPKC (1/1000, Santa Cruz Biotechnology), rabbit anti-Par3 (1/1000, Millipore) and rabbit anti-PALS1 (1/1000, Santa Cruz Biotechnology). HRP-conjugated secondary antibodies were from GE Healthcare and were used at a 1/1000 dilution.

### RT-PCR/qPCR analysis

RNA was extracted with TRizol (Invitrogen) according to manufacturer’s instructions. 2 μg of total RNA was reverse transcribed using the Superscript II Reverse Transcriptase kit (Invitrogen) as prescribed by the company. qPCRs were performed using the FastStart Universal SYBR Master (Rox) (Roche). Samples were normalized to actin F4 (5′-GAT CAT CCG CAA GCC TGT GA -3′) and R4 (5′-GCA TCC GAG GAT TGG CAG TA -3′), and fold change was calculated by the 2^−ΔΔCt^ method. *Crb3* cDNA was amplified with the following forward (5′-ATG GCG ACC CCA GGC CTG- 3′) and reverse (5′-AGG CCC CCG TTG GAC TCA T-3′) primers, whereas the following primer pair was used for *Crb2* (forward 5′-AGCATGGATGTGGATGAGTG-3′; reverse 5′- GCTGCACAGAACAGTCATGT-3′).

### Statistical analysis

Statistical significance was assessed using the Student’s *t* test or ANOVA analysis of variance. A *P* value inferior to 0.05 was considered statistically significant.

## Additional Information

**How to cite this article**: Charrier, L. E. *et al*. Mouse Crumbs3 sustains epithelial tissue morphogenesis *in vivo*. *Sci. Rep.* **5**, 17699; doi: 10.1038/srep17699 (2015).

## Supplementary Material

Supplementary Information
