# Supplementary material for: Mouse Crumbs3 sustains epithelial tissue morphogenesis in vivo
Source: Sci Rep. 2015 Dec 3;5:17699. doi: 10.1038/srep17699 (PMC4668553; doi:10.1038/srep17699)
Supplement: Supplementary Information [file srep17699-s1.pdf]

Mouse Crumbs3 sustains epithelial tissue morphogenesis *in vivo*

Lucie E. Charrier[a,b,], Elise Loie[a,b] and Patrick Laprise[a,b,#]

Département de Biologie Moléculaire, Biochimie Médicale et Pathologie/Centre de Recherche sur le Cancer, Université Laval[a], and CRCHU de Québec-axe oncologie[b], Québec, Canada.

[#]Address correspondence to Patrick Laprise, Patrick.Laprise@crchudequebec.ulaval.ca.

Supplementary figures 1 to 5

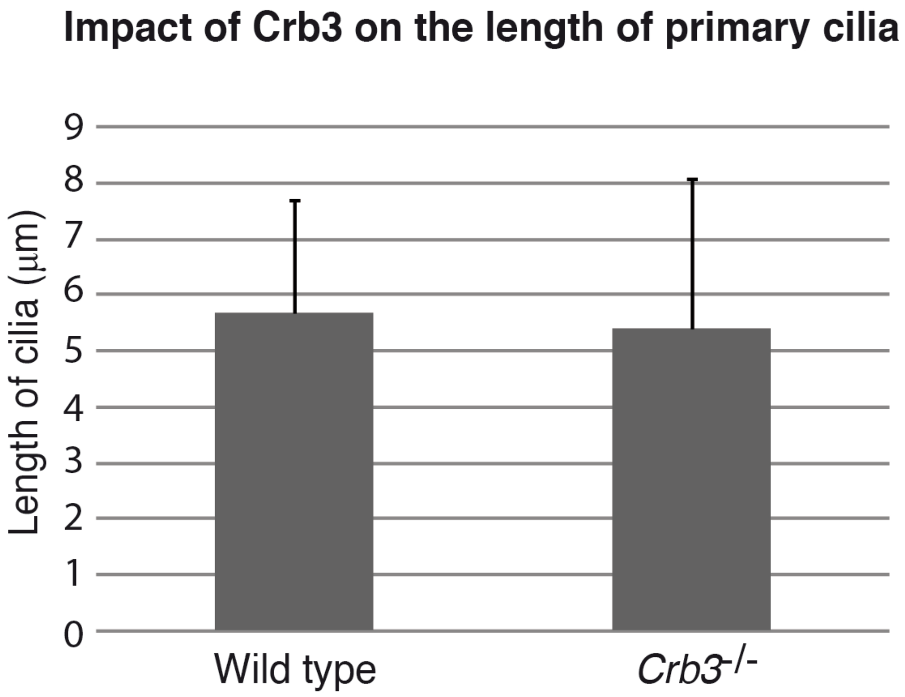

Supplementary Figure 1 | Histogram showing the average length of primary cilia at the surface of kidney epithelial cells (n = 100) of wild type and *Crb3*$^{-/-}$ embryos (E18.5). Normal and Crb3-deprived cells form a primary cilium of comparable length.

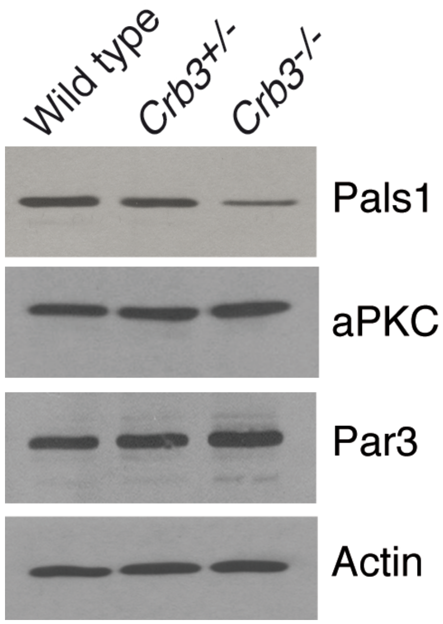

Supplementary Figure 2 | Western blot analysis of Pals1, aPKC and Par3 in kidneys of control, *Crb3*$^{+/-}$ and *Crb3*$^{-/-}$ embryos. Actin was used as loading control. Loss of Crb3 is associated with a decrease in Pals1 levels. However, the level of aPKC and Par3 is not affected by the loss of Crb3.

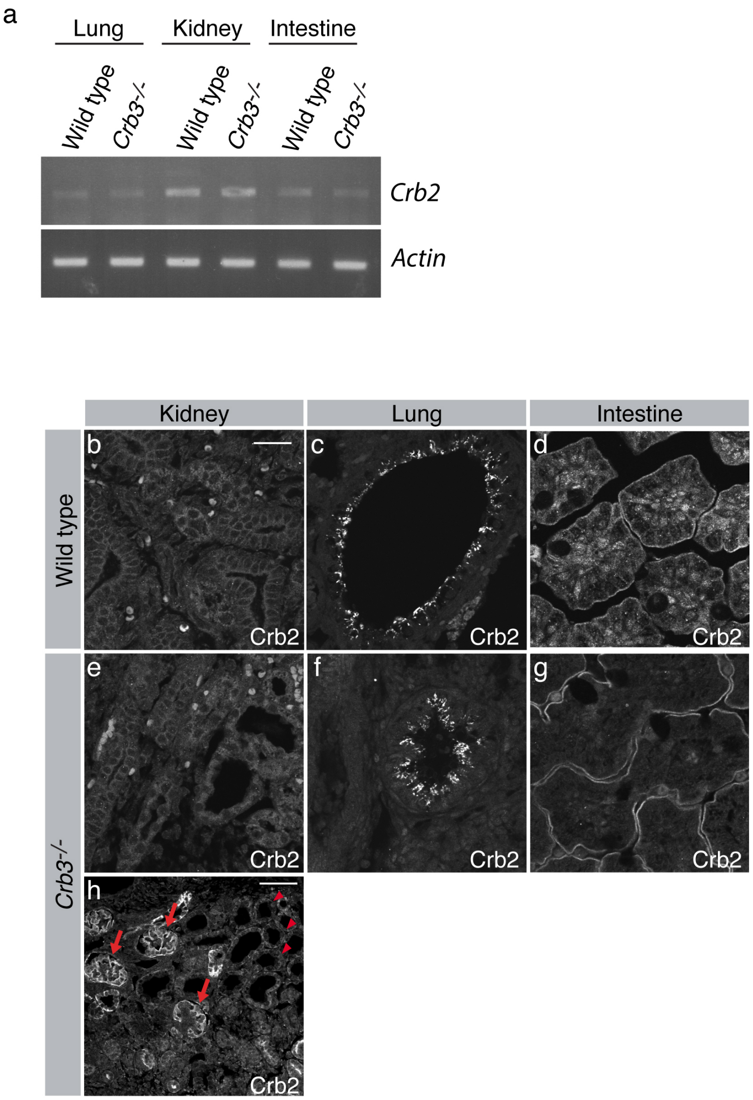

Supplementary Figure 3 | Crb2 distribution in kidney and lung epithelia. (a) RT/semi-quantitative PCR analysis of *Crb2* expression in lungs, kidneys and intestine of E18.5 wild type embryos or *Crb3* homozygous mutant embryos (*Crb3*$^{-/-}$). (b-h) Paraffin-embedded lung, kidney and intestinal histological sections of wild type or *Crb3* knockout (*Crb3*$^{-/-}$) were stained for Crb2. Scale bar in b represents 20 μm and also applies to c-g. Scale bar in h represents 40 μm.

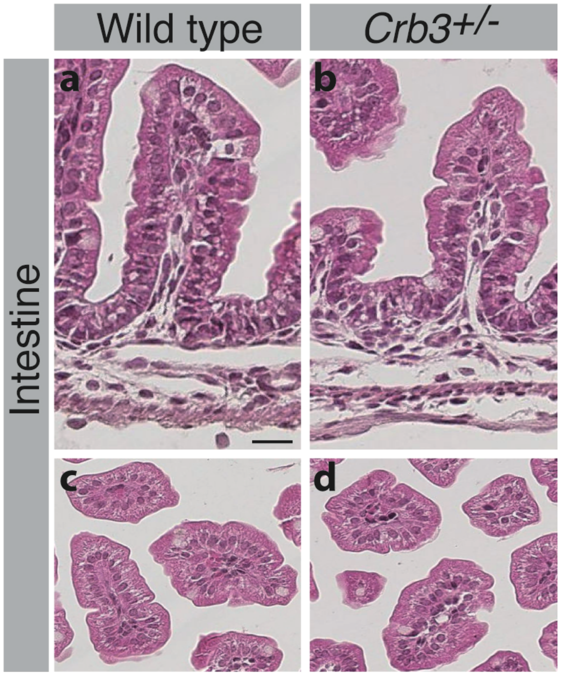

Supplementary Figure 4 | Paraffin-embedded intestine of wild type or *Crb*$^{+/-}$ mouse embryos (E18.5) were sectioned and stained with Hematoxylin and Eosin (H&E).

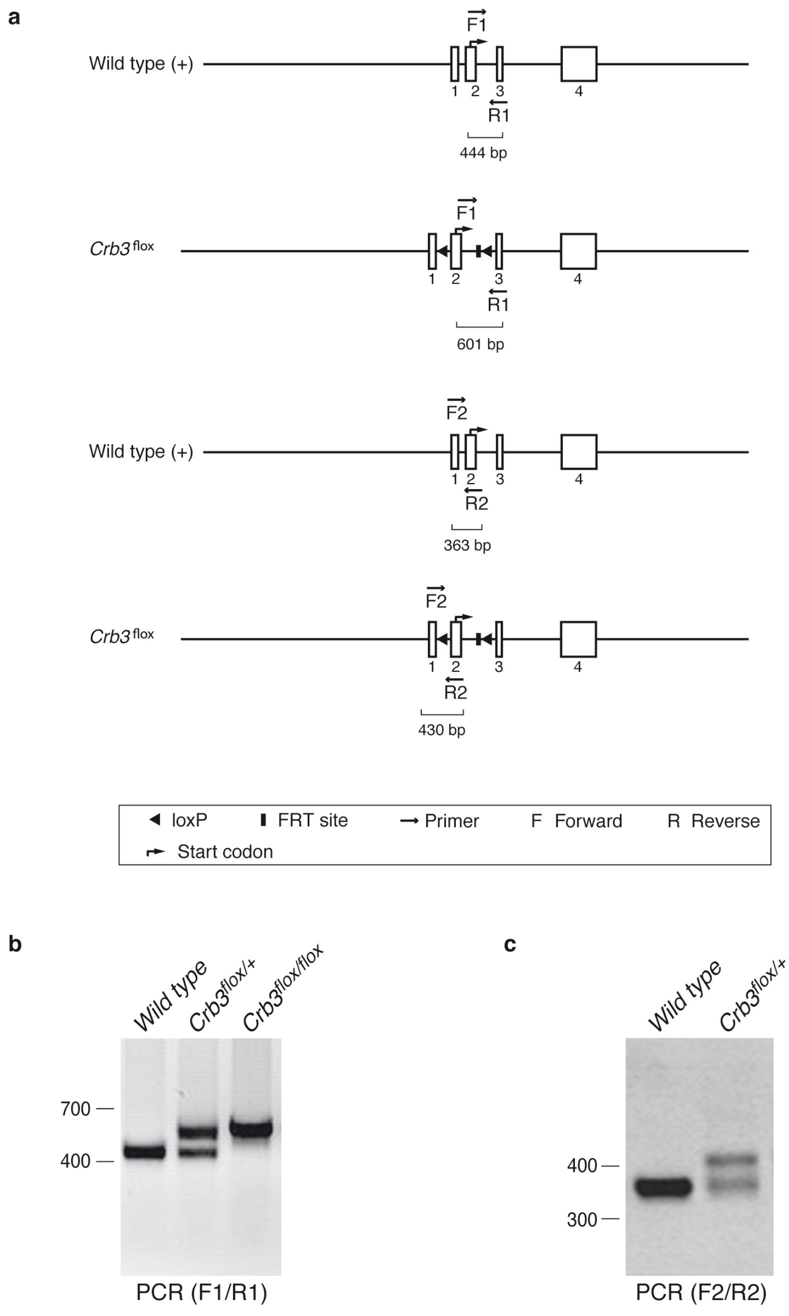

Supplementary Figure 5 | Genotyping of the *Crb3* floxed allele by PCR. (a) Schematic representation of the PCR strategies used to discriminate the wild type and *Crb3*^{flox} alleles. (b and c) Representative PCR reactions using the F1/R1 (b) or F2/R2 (c) primer pairs allowing to detect loxP sites flanking exon 2 in mice carrying the *Crb3*^{flox} allele.
